# Mesenchymal Stem Cells from Cervix and Age: New Insights into CIN Regression Rate

**DOI:** 10.1155/2018/1545784

**Published:** 2018-12-02

**Authors:** Monia Orciani, Miriam Caffarini, Raffaella Lazzarini, Giovanni Delli Carpini, Dimitrios Tsiroglou, Roberto Di Primio, Andrea Ciavattini

**Affiliations:** ^1^Department of Clinical and Molecular Sciences-Histology, Università Politecnica delle Marche, Via Tronto 10/a, 60126 Ancona, Italy; ^2^Clinic of Obstetrics and Gynecology, Department of Clinical Sciences, Università Politecnica delle Marche, Via Tronto 10/a, 60126 Ancona, Italy

## Abstract

Cervical intraepithelial neoplasia (CIN) is a precancerous lesion of the uterine cervix that can regress or progress to cervical cancer; interestingly, it has been noted that young women generally seem to have higher rates of spontaneous regression and remission, suggesting a correlation between the patient's age and regression/progression rates of CIN. Even if the underlying mechanisms are still unclear, inflammation seems to play a pivotal role in CIN fate and inflammatory processes are often driven by mesenchymal stem cells (MSCs). This study was aimed at evaluating if age affects the behavior of MSCs from the cervix (C-MSCs) that in turn may modulate inflammation and, finally, regression rate. Fourteen samples of the human cervix were recovered from two groups of patients, “young” (mean age 28 ± 2) and “old” (mean age 45 ± 3), during treatment using the loop electrosurgical excision procedure (LEEP) technique. Progenitor cells were isolated, deeply characterized, and divided into young (yC-MSCs) and old cervixes (oC-MSCs); the senescence, expression/secretion of selected cytokines related to inflammation, and the effects of indirect cocultures with HeLa cells were analyzed. Our results show that isolated cells satisfy the fixed criteria for stemness and display age-related properties; yC-MSCs express a higher level of cytokines related to acute inflammation than oC-MSCs. Finally, in the crosstalk with HeLa cells, MSCs derived from the cervixes of young patients play a stronger antitumoral role than oC-MSCs. In conclusion, the immunobiology of MSCs derived from the cervix is affected by the age of donors and this can correlate with the regression rate of CIN by influencing their paracrine effect. In addition, MSCs from a young cervix drives an antitumoral effect by sustaining an acute inflammatory environment.

## 1. Introduction

High-grade cervical intraepithelial neoplasia (CIN2–3) is a precancerous lesion of the uterine cervix with an incidence of 250,000–1 million annually in the USA. CIN can either resolve spontaneously or persist or progress to cervical cancer when not treated immediately [1]. High-grade CIN are typically treated with cervical excision; since this procedure may correlate with an increased risk for prematurity and adverse pregnancy outcomes [2–4], each patient needs an individual evaluation, especially in the case of young women with a desire for future pregnancy, with a careful selection of patients who can be treated surgically and those who can be managed conservatively, as well as with the consideration of the possibility of a regression of the lesions [5].

Interestingly, several researches [1, 6, 7] have evaluated the possible correlation between a patient's age and regression/progression rates of CIN, finding that younger women generally seem to have higher rates of spontaneous regression and remission. The underlying mechanisms are not yet fully known but, as for other solid tumors, inflammation may play a pivotal role in CIN fate [8]. The hypothesis of an involvement of inflammation in CIN progression is enforced also by studies aimed at evaluating the use of anti-inflammatory drugs in the treatment of CIN [9].

Inflammation is orchestrated by different cells of the immune system as well as by mesenchymal stem cells (MSCs) [10]; the existence of MSCs in cervical tissue has been strongly strengthened by the evidence that, within six months of the LEEP, the cervix shows a regenerative process, reporting values of 71–98% of postexcision tissue deficiency [11–13]. MSCs from the cervix (C-MSCs) have been previously characterized [14]; here, we deepen this characterization by comparing the properties of MSCs isolated from the cervix of women of different ages to understand if MSCs show age-related functions and if immunobiology is able to explain the different regression rates observed in young/old women. In addition, since CIN may progress to cervical cancer, MSCs isolated from cervixes of young and old patients were cocultured with a cervical tumoral cell line (HeLa) and their effects were tested.

## 2. Material and Methods

### 2.1. Patient Enrollment

Fourteen women undergoing cervical excision for high-grade CIN, diagnosed after colposcopy-directed cervical punch biopsy following an abnormal cervical cytology, were enrolled in this study; seven patients were defined “young” (mean age 28 ± 2), and the others were defined “old” (mean age 45 ± 3). Patients provided their written informed consent to participate in the study, which was approved by the institutional ethics committees and was conducted in accordance with the Declaration of Helsinki.

### 2.2. Cervical Excision

The cervical excisional procedures were performed with the loop electrosurgical excision procedure (LEEP) technique, in an outpatient setting under local anesthesia and strict colposcopic guidance, with 1.5–2.0 cm rounded loops, chosen according to the type of the transformation zone and the area of cervical tissue to remove.

### 2.3. Cell Culture

Tissue fragments (2–3 mm^3^), obtained from cone specimen, were placed into 6-well plates containing MSCGM medium (Lonza, Basel, Switzerland), as previously described [15–18]. Cell morphology was evaluated by phase-contrast microscopy (Leica DM IL; Leica Microsystems GmbH, Wetzlar, Germany), and viability by an automated cell counter (Invitrogen, Milano, Italy). All further analyses involved separate assays of the specimens from the fourteen participants up to the first five passages. MSCs isolated from the cervixes of young patients were named yC-MSCs, while MSCs isolated from cervixes of old patients were defined oC-MSCs. All the subsequent experiments were performed with y- and oC-MSCs at the same passage of culture.

### 2.4. Characterization of Mesenchymal Stem Cells from Young and Old Cervixes

According to the criteria identified by Dominici et al. [19], cells were characterized by testing the plastic adherence, immunophenotype, and multipotency.

For immunophenotyping, 2.5 × 10^5^ cells were stained for 45 min with fluorescein isothiocyanate- (FITC-) conjugated antibodies (Becton Dickinson) against HLA-DR, CD14, CD19, CD34, CD45, CD73, CD90, and CD105. In addition, the expression of CD9 was analyzed as a distinguishing marker between MSCs and fibroblasts.

For the differentiation assay, cells were induced towards osteocytes, chondrocytes, and adipocytes using StemPro® Osteogenesis, Chondrogenesis, and Adipogenesis Kits (Gibco, Invitrogen), respectively [20]. Osteogenic differentiation was assessed by Von Kossa and alkaline phosphatase (ALP) staining after 10 days; adipogenic differentiation was tested by Oil Red staining after 15 days. For chondrogenesis, cells were cultured in a pellet culture system for 30 days, and the sections were exposed to a solution of Safranin-O. Cells cultured in MSCGM alone were used as negative controls. Finally, the expression of genes related to stemness (OCT4, SOX2, NANOG, and KFL4) was analyzed by flow cytometry (as above reported, antibodies against SOX2, NANOG, and KLF4 from Invitrogen and OCT4 from Sigma-Aldrich) and by RT-PCR.

For RT-PCR, total RNA isolation, reverse transcription, and amplification were as previously described [21]. mRNA expression was calculated by the 2^−ΔΔ*C*_t_^ method [22], where Δ*C*_t_ = *C*_t_(gene of interest) − *C*_t_(control gene) and Δ(Δ*C*_t_) = Δ*C*_t_(yC − MSCs) − Δ*C*_t_(oC − MSCs). The values of the relative expression of the genes are mean ± standard deviation (SD) of three independent experiments. Primer sequences are reported in Table 1.

### 2.5. Proliferation Assay

To assess the proliferation rate, the XTT Cell Proliferation Assay (Trevigen, Gaithersburg, MD, USA) was performed on cells after 72 hours of culture for the first 6 passages. The data about yC-MSCs are reported as percentages of the values measured in parallel in oC-MSCs (referred to as 100%) over three independent experiments.

### 2.6. Senescence-Associated *β*-Galactosidase Assay

SA-*β*-Gal activity was detected with a senescent cell staining kit (Sigma-Aldrich, Milan, Italy) according to the manufacturer's instructions.

Briefly, 7 × 10^4^ cells at passage 6th were seeded in a six-well plate and incubated overnight with the staining solution. *β*-Gal was microscopically revealed by the presence of a blue, insoluble precipitate within the cell, and the percentage of SA-*β*-Gal-positive cells was determined by counting at least 500 cells in each sample.

### 2.7. PCR Array for the Senescence

The expression of 86 genes related to senescence was analyzed by PCR array (Qiagen, Milan) in MSCs isolated from cervixes derived from young and old patients.

Total RNA was isolated by using Masterscript RT-PCR System (5 PRIME, Hamburg, Germany). cDNA synthesis was performed using SABiosciences RT^2^ First Strand Kits, following the manufacturer's instruction. mRNA expression was calculated by the 2^−ΔΔ*C*_t_^ method [22], where Δ*C*_t_ = *C*_t_(gene of interest) − *C*_t_(control gene) and Δ(Δ*C*_t_) = Δ*C*_t_(yC − MSCs) − Δ*C*_t_(oC − MSCs).

### 2.8. Culture of HeLa

The cell line HeLa (human cervix epithelioid carcinoma from Sigma-Aldrich) was cultured in EMEM (EBSS) + 2 mM glutamine + 1% nonessential amino acids (NEAA) + 10% foetal bovine serum (Sigma-Aldrich).

### 2.9. Cocultures of C-MSCs and HeLa Cells and Proliferation Analysis

HeLa cells were cocultured with MSCs to evaluate the paracrine effects exerted by stem cells isolated from cervixes of young and old patients.

5 × 10^4^ HeLa cells were seeded at the lower surface, and 5 × 10^4^ of yC- or oC-MSCs were individually added the day after at the upper surface of a polycarbonate transmembrane filter in a Transwell filter system in a 6-well plate (pore size 0.4 *μ*m; BD Falcon). Cells were cocultured for 72 hours. After cocultures, tumor cells were recovered and their number was assessed by an automated cell counter. HeLa cells cultured individually served as controls (mock). Data are reported as mean ± SD from three independent experiments.

### 2.10. Expression of Selected Genes in Cocultured HeLa Cells

RNA was isolated from HeLa cells, mock, and after individual cocultures with MSCs derived from young and old cervixes. The genes and the related primer sequences are summarized in Table 1; these genes are associated with specific cellular mechanisms, such as oncogenesis (cMET, cFOS, and cJUN), proliferation (mKI67), invasion and migration (MMP11), and angiogenesis (VEGF, CXCL12). All samples were tested in triplicate with the reference genes GAPDH, *β*-actin, and RPE30 for data normalization. Of the three, GAPDH was chosen since it was the most stable one.

Quantification of mRNA expression was calculated with the 2^−ΔΔ*C*_t_^ method, where Δ*C*_t_ = *C*_t_(gene of interest) − *C*_t_(control gene) and Δ(Δ*C*_t_) = Δ*C*_t_(HeLa cells cocultured with C − MSCs) − Δ*C*_t_(HeLa cells cultured alone). The values of the relative expression of genes of interest are referred to as mean ± SD over three independent experiments.

### 2.11. Expression of Inflammation-Related Cytokines by ELISA Test

Selected cytokines related to acute and chronic inflammation, such as IL6, IL12, IFN-*γ*, TNF-*α*, IL2, IL4, IL5, IL13, IL10, TGF-*β*, IL17A, and G-CSF, were investigated by ELISA (Multi-Analyte ELISArray Kit, Qiagen, Milan, Italy) as previously described [23]. The analysis was firstly performed on MSCs derived from cervixes of young and old patients to assess potential age-related differences in the immunobiology of MSCs. Mean ± SD has been calculated for yC-MSCs and for oC-MSCs over three independent tests and is expressed as pg/ml. Subsequently, MSCs were cocultured with HeLa cells and the amount of the differentially secreted cytokines was reevaluated. Levels detected in cocultured HeLa have been reported as a percentage of the levels measured in Hela cells cultured alone, and data are presented as mean ± SD over three independent tests.

### 2.12. Statistical Analysis

Statistical analysis of data from at least 3 independent experiments was performed using SPSS 19.0 software (SPSS Inc., Chicago, IL, USA). All data are mean ± SD. For two-sample comparisons, significance was calculated by Student's *t*-test using SPSS 17.0 software. *p* values ≤ 0.05 were considered significant.

## 3. Results

MSCs were successfully isolated from the cervixes of all patients, subgrouped into “young” and “old.” No statistically relevant difference was found in each cellular group among the donors. Therefore, results are reported as mean ± SD for young and old patients in each analysis. For all the subsequent experiments, yC-MSCs and oC-MSCs were used at the same passage of culture.

### 3.1. Cell Isolation and Characterization

**Figure 1:**
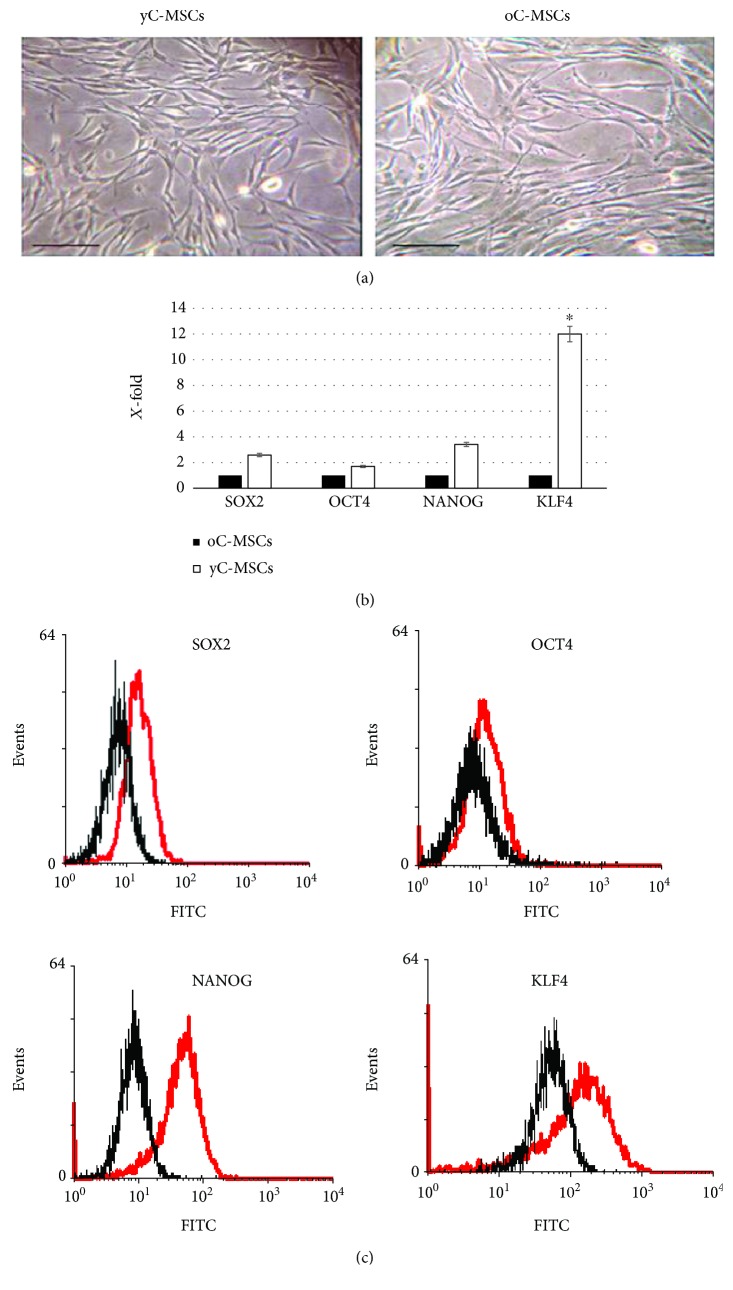
Morphology and expression of genes related to stemness. (a) Phase-contrast images of MSCs derived from the cervixes of younger (yC-MSCs, left) and older patients (oC-MSCs, right) after 14 days of culture. Scale bar, 100 *μ*m. The expression of selected markers related to self-renewal and differentiation potential (OCT4, SOX2, NANOG, and KLF4) was evaluated by RT-PCR (b) and cytofluorimetry (c). (b) Levels of expression detected in yC-MSCs are referred to as *X*-fold with respect to oC-MSCs (expressed as equal to 1). Data are means ± SD from analyses performed on three separate experiments in triplicate. ^∗^*p* < 0.05 for yC-MSCs versus oC-MSCs. (c) Representative FACScan analyses of cell-surface antigen expression, as indicated. Black histograms refer to oC-MSCs and red histograms to yC-MSCs.

Cell cultures (from cervixes of young and old patients) showed a fibroblastoid morphology (Figure 1(a)).

Both cell types expressed all the stemness genes tested by RT-PCR, with a higher expression in cells derived from the cervixes of young patients. Considering as 1 the expression detected in MSCs from old cervixes, the same genes are from 1.74 ± 0.21 (OCT4) up to 12.05 ± 0.53 (KLF4) fold higher in MSCs from young patients (Figure 1(b)). This trend was confirmed at the protein level, as revealed by cytofluorimetric analysis (Figure 1(c)).

Evaluation of the stemness criteria identified by Dominici et al. demonstrated that cells were strongly positive for CD73, CD90, and CD105 and negative for HLA-DR, CD14, CD19, CD34, and CD45, as well as for the key marker CD9 (Table 2).

**Figure 2:**
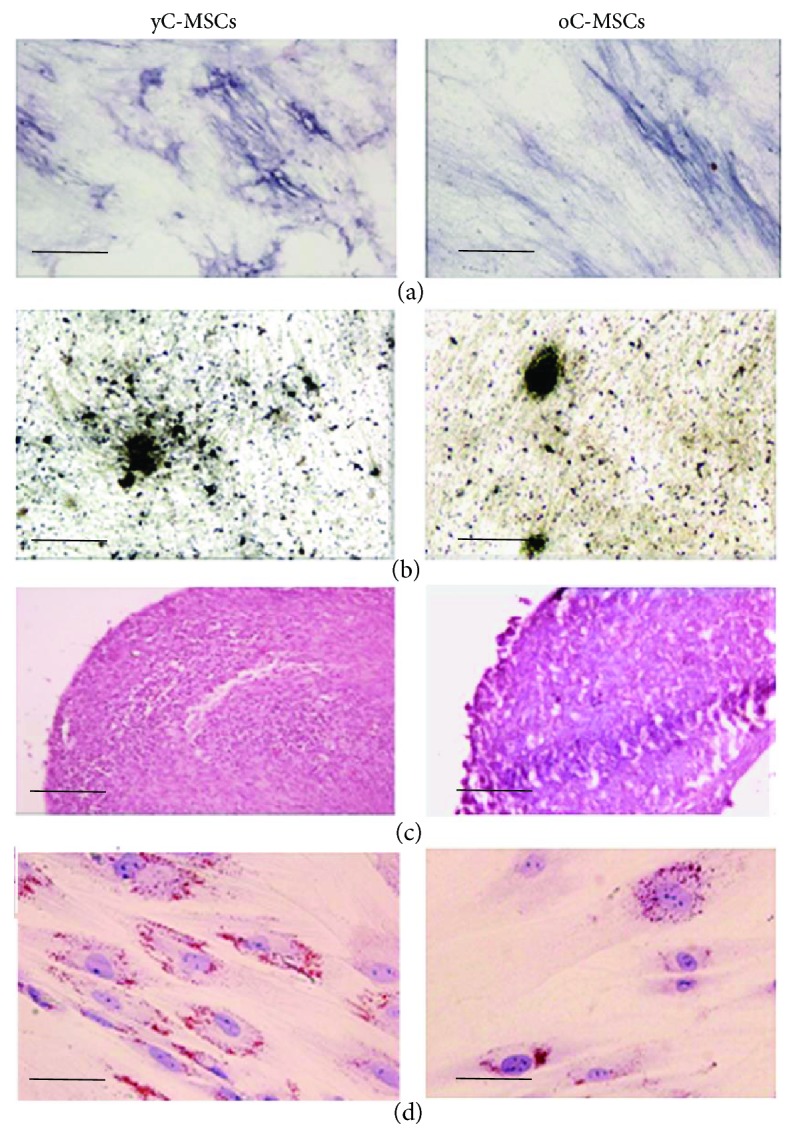
Multilineage differentiation of MSCs derived from younger (yC-MSCs) and older (oC-MSCs) cervixes. Representative images of osteogenic differentiation assessment by ALP reaction (a, scale bar 100 *μ*m) and Von Kossa staining (b, scale bar 100 *μ*m); chondrogenic differentiation by Safranin-O coloration (c, scale bar 200 *μ*m); and adipocyte differentiation by Oil Red staining (d, scale bar 30 *μ*m).

Cells were also capable of differentiating towards osteogenic, chondrogenic, and adipogenic lineages (Figure 2).

**Figure 3:**
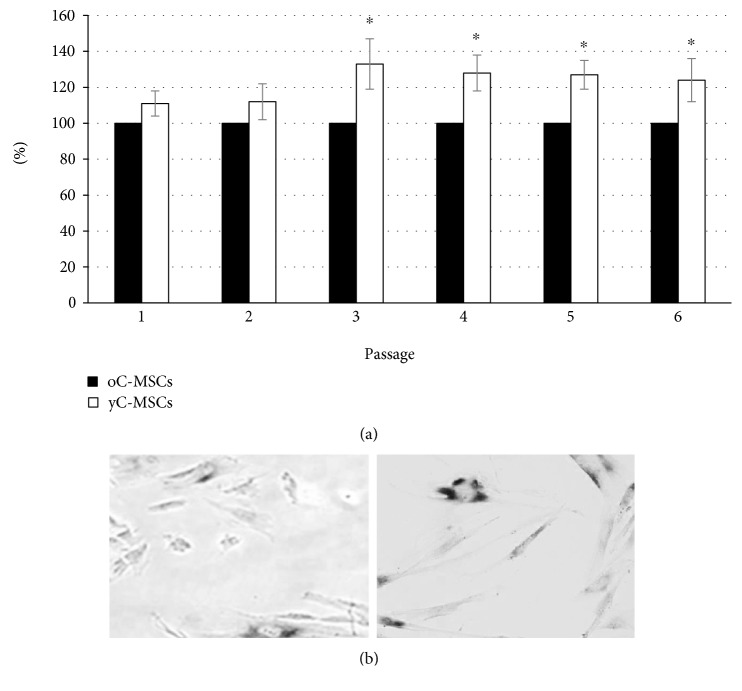
XTT assay and senescence-associated beta-galactosidase assays of MSCs derived from younger (yC-MSCs) and older (oC-MSCs) cervixes. (a) XTT assay performed after 72 hours of culture for the first 6 passages. Data from oC-MSCs are expressed as 100%, and those for yC-MSCs are calculated accordingly. Data are reported as mean ± SD of three independent experiments. ^∗^*p* < 0.05 for yC-MSCs versus oC-MSCs. (b) Representative micrographs obtained with phase-contrast microscopy showing SA-*β*-Gal activity in cells at passage 6.

XTT assay was performed after 72 hours of cultures of y- and oC-MSCs for the first six passages (Figure 3(a)). yC-MSCs displayed a higher proliferation rate than oC-MScs (referred to as 100%).

Looking at the data from the proliferation assay and with the aim of assessing potential differences between MSCs derived from cervixes of young and old patients, cellular senescence was tested.

Senescence was evaluated in yC-MSCs and oC-MSCs at passages 6 and interestingly the number of blue cells (indicating senescence) was notably different between yC-MSCs and oC-MSCs (11% and 23%, respectively) (Figure 3(b)).

### 3.2. PCR Array for Senescence in oC-MSCs and yC-MSCs

**Figure 4:**
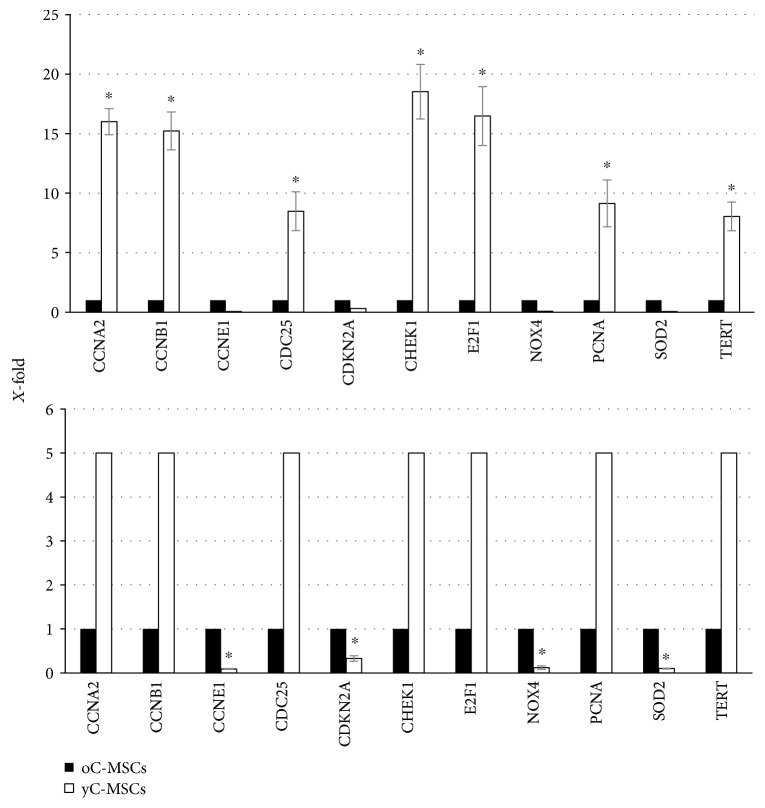
Expression of genes related to senescence. The expression of 86 genes referred to senescence was evaluated by a PCR array. Choosing a 3-fold cut-off, expression of CCNA2, CCNB1, CDC25, CHEK1, E2F1, TERT, and PCNA was upregulated in yC-MSCs compared to oC-MSCs, while that of CDKN2A, CCNE1, NOX4, and SOD2 was downregulated. Data are means ± SD from analyses performed on three separate experiments. ^∗^*p* < 0.05 for yC-MSCs versus oC-MSCs.

The expression of more than 80 genes related to senescence was analyzed by PCR and expressed as 1 in oC-MSCs and accordingly as *X*-fold in yC-MSCs. The expression of all genes revealed a dysregulation in yC-MSCs compared to oC-MSCs. A 3-fold cut-off was chosen for the significance; the expression of CCNA2, CCNB1, CDC25, CHEK1, E2F1, TERT, and PCNA was significantly higher in yC-MSCs than in oC-MSCs, while that of CDKN2A, CCNE1, NOX4, and SOD2 was downregulated in yC-MSCs compared to oC-MSCs (Figure 4).

### 3.3. Proliferation of HeLa Cells after Cocultures with MSCs

**Figure 5:**
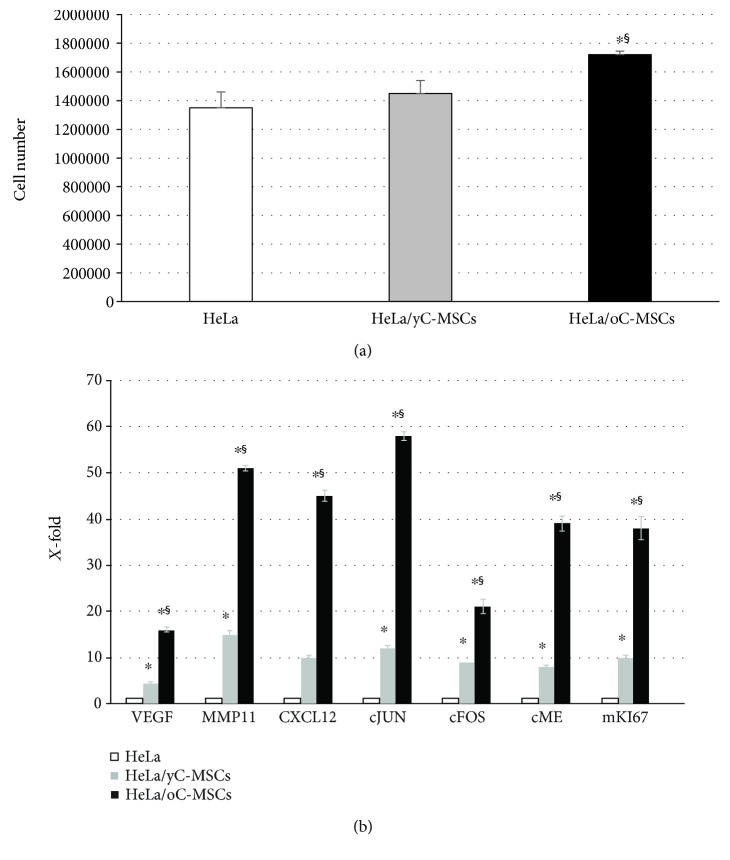
Proliferation and expression of genes related to tumorigenesis in HeLa cells after indirect coculture with MSCs. HeLa cells were indirectly cocultured for 72 hours with yC-MSCs (HeLa/yC-MSCs) or oC-MSCs (HeLa/oC-MSCs). HeLa cultured alone were used as control. (a) Proliferation was assessed by an automated cell counter; ^∗^*p* < 0.05 for HeLa/yC-MSCs or HeLa/oC-MSCs versus HeLa and ^§^*p* < 0.05 for HeLa/yC-MSCs versus HeLa/oC-MSCs. (b) The histogram displays the expression of selected genes related to specific cellular mechanisms, such as oncogenesis (cFOS, cJUN, and cMET), invasion and migration (MMP11), proliferation (mKI67), and angiogenesis (VEGF, CXCL12) in HeLa cells after cocultures with the yC-MSCs and oC-MSCs. Levels of expression detected in cocultured HeLa are referred to as *X*-fold with respect to individually cultured HeLa (expressed as 1). ^∗^*p* < 0.05 for HeLa/yC-MSCs or HeLa/oC-MSCs versus HeLa and ^§^*p* < 0.05 for HeLa/yC-MSCs versus HeLa/oC-MSCs.

Tumor cells were recovered from cocultures and cell number assessed by an automated cell counter. Compared with the mock sample, only HeLa cells cocultured with MSCs from cervixes of old women had a significantly (*p* < 0.05) higher proliferation rate (Figure 5(a)).

### 3.4. Gene Expression

The expression of selected genes was analyzed by RT-PCR in HeLa cells before (mock) and after cocultures with yC-MSCs and oC-MSCs. Compared to mock HeLa (calculated as 1), cocultured HeLa exhibited significantly (*p* < 0.05) higher levels of cMET, cFOS, cJUN, MMP11, CXCL12, and VEGF. These variations were significantly (*p* < 0.05) more evident in HeLa cocultured with oC-MSCs than with yC-MSCs (Figure 5(b)).

### 3.5. Expression Profile of Inflammatory Cytokines

The secretion of inflamed-related cytokines was evaluated by ELISA.

**Figure 6:**
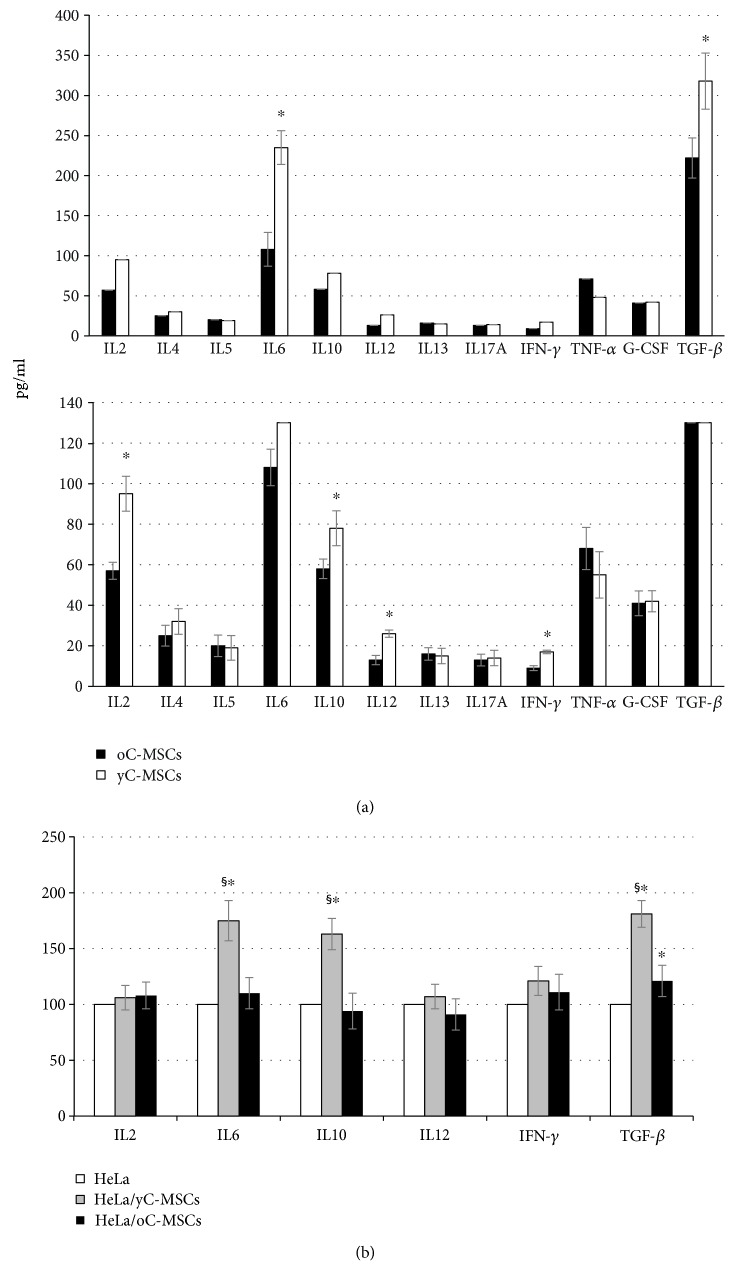
Detection of secreted cytokines in yC-MSCs and oC-MSCs by ELISA before (a) and after cocultures with HeLa cells (b). (a) Level of secretion of selected cytokines referred to inflammation detected in MSCs from young (yC-MSCs) and old (oC-MSCs) cervixes. Data, expressed in pg/ml, are means ± SD from analyses performed on three separate experiments. (b) Histograms displaying the levels of the same cytokines detected after indirect cocultures with HeLa cells for 72 hours. The level of each cytokine is shown as % (mean ± SD from three independent experiments) of the level detected in control HeLa cells. ^∗^*p* < 0.05 for HeLa/yC-MSCs or HeLa/oC-MSCs versus HeLa and ^§^*p* < 0.05 for HeLa/yC-MSCs versus HeLa/oC-MSCs.

Firstly, the detection was performed in yC-MSCs and oC-MSCs (Figure 6(a)) to identify potential age-related differences. Independently from age, the most secreted cytokines were IL6 and TGF-*β*, followed by IL2, IL10, and TNF-*α*. Compared to oC-MSCs, MSCs from cervixes of young patients exhibited significantly (*p* < 0.05) higher levels of IL2, IL6, IL10, IL12, IFN-*γ*, and TGF-*β*. The expression of IL4, IL5, IL13, IL17A, TNF-*α*, and G-CSF was not significantly different between oC-MSCs and yC-MSCs. Subsequently, C-MSCs were cocultured with HeLa cells and the secretion of the differently expressed cytokines was measured in HeLa mock (expressed as 100%) and in cocultured HeLa (accordingly calculated). Compared to the mock sample, HeLa cocultured with yC-MSCs showed a significantly (*p* < 0.05) increased secretion of IL6, IL10, and TGF-*β*, where the production of IL2, IL12, and IFN-*γ* did not change. Cocultures with oC-MSCs produce a significant increase only in the secretion of TGF-*β* (Figure 6(b)).

## 4. Discussion

The annual regression rate of CIN2 in old women is estimated to range from 15 to 23%, while in young women the regression rate is 65% [24]; a lower regression rate (6–38%) is instead reported for CIN3 [25, 26]. Inflammation, sustained also by undifferentiated mesenchymal stem cells (MSCs) [10], plays a pivotal role in the progression or regression of the CIN. The existence of a reservoir of MSCs inside the cervix (C-MSCs) is well accepted and they are considered also the promoters of cervical regeneration after LEEP. May MSCs display age-related features able to explain the different regression rate in young and old women? And in case of progression towards cervical tumor, what is the role of MSCs and their correlation with age?

Firstly, fourteen patients were enrolled in this study; 7 women were defined as “young” (mean age 28 ± 2), and the others were defined as “old” (mean age 45 ± 3). From all tissue biopsies, it was possible to isolate an undifferentiated cellular population able to satisfy all the minimal criteria to qualify as mesenchymal stem cells. Cells isolated from young cervixes were named yC-MSCs and those from old cervixes oC-MSCs. Both yC-MSCs and oC-MSCs were cultured as plastic adherent, were negative for CD14, CD19, CD34, CD45, and HLA-DR, and were positive for CD73, CD90, and CD105. Cells were also able to differentiate towards adipogenic, chondrogenic, and osteogenic lineages. These properties were equally displayed by yC-MSCs and oC-MSCs without evident differences. The subsequent analysis of the expression of genes related to stemness, such as OCT4, SOX2, NANOG, and KLF4, revealed the first discrepancy between young and old MSCs. yC-MSCs express a higher value of KLF4 than oC-MSCs. KLF4 directly binds to the promoter of NANOG to help OCT4 and SOX2 in regulating the expression of NANOG [27]. This observation confirms the critical role of KLF4 in stem cell self-renewal as well as pluripotency. Another evident difference between yC-MSCs and oC-MSCs was related to the proliferation rate that was faster in yC-MSCs than in oC-MSCs. To clarify the possible involvement of senescence, this mechanism was investigated by *β*-galactosidase assay. The analysis confirmed that at passage 6 MSCs derived from the cervixes of old patients display a higher percentage of senescent cells than MSCs from young patients. Subsequently, the expression of genes related to senescence was tested by PCR array. Choosing a 3-fold cut-off, the expression of 13 genes was dysregulated between MSCs derived from young and old cervixes; in detail, the expression of CCNA2, CCNB1, CDC25, CHEK1, E2F1, TERT, and PCNA was upregulated in yC-MSCs compared to oC-MSCs, while that of CDKN2A, CCNE1, NOX4, and SOD2 was downregulated. The observed dysregulations reflect the role of these genes in senescence [28]. CCNA2 is fundamental for cell proliferation [29]. TERT plays a pivotal role in telomerase length, and its expression is higher in dividing cells and gradually decreases in senescent cells [30–32].

E2F1 and CHEK1 regulate, respectively, the expression of genes referred to cell cycle progression and the G2 DNA damage checkpoint [33, 34] and are both less expressed in senescent MSC [35]. A downregulation of CDC25C is associated with cell cycle arrest [36], more precisely in the phase G2/M [37]. Finally, the expression of CCNB1 is lower in senescent MSCs, as already reported by Noh et al. [35], as well as the expression of PCNA that codes the proliferating cell nuclear antigen expressed exclusively in actively proliferating cells [38].

Among the downregulated genes in yC-MSCs, CCNE1 [39] and CDKN2A are known to be upregulated in senescent cells compared to early-passage cells and play a role in achieving the senescent state [40, 41]; NOX4, a member of the NADPH oxidase family, increased ROS production and is related to senescence [42]; the overexpression of SOD2 in oC-MSCs is in agreement with Estrada et al. [43] and confirms that senescence significantly alters MSC metabolism and oxidative status.

To test if the involvement of inflammation in CIN progression could be age-related, the secretion of soluble factors related to acute and chronic inflammation was analyzed by ELISA in y- and oC-MSCs. The first evidence is the increased secretion of cytokines related to the acute inflammation (IL2, IL6, IL10, IL12, IFN-*γ*, and TGF-*β*1) in MSCs from cervixes of young patients. Previous studies have suggested that decreased Th1 responses (strictly connected to acute inflammation) are associated with cervical carcinogenesis [44]. This different ability of MSCs to secrete cytokines may correlate with the altered age-related regression rate. Acute inflammation allows the organism to counteract infection, such as the infection with oncogenic human papillomaviruses (HPV) that plays a central etiologic role in the development of squamous carcinomas of the cervix and their precursor lesions like CIN. The profile of cytokines secreted by MSCs derived from old cervixes seems to indicate that the cervical microenvironment is not able to respond with an acute inflammation, with a subsequent incapacity to counteract HPV and CIN as demonstrated by the lower percentage of regression.

If CIN does not regress, it may develop towards cervical carcinogenesis.

As for other tumors, inflammation appears to exert a major effect. If the condition causing acute inflammation is not resolved, the inflammation may become chronic, favoring tumor onset and development. Following this hypothesis, MSCs from cervixes of old and young patients were indirectly cocultured with a cervix epithelioid carcinoma cell line (HeLa). Firstly, it was assessed if factors secreted by MSCs may alter the proliferation of tumor cells, finding that cocultures with oC-MSCs produce an increase in HeLa proliferation rate. Subsequently, the expression of genes related to different mechanisms leading to tumor development was assayed in control and cocultured HeLa cells. PCR analysis revealed that, after cocultures with C-MSCs, HeLa cells expressed increased levels of all the selected genes and the observed increase was significantly higher in cocultures with MSCs derived from old than from young cervixes. In detail, the upregulation of cJUN, cFOS, and cMET after cocultures especially with oC-MSCs correlates with other works that underlined that these genes are implicated in cervical carcinogenesis [45–47]. VEGF and CXCL12 are master regulators of neoangiogenesis that drives tumor development. MMP11 is related to invasion and migration, two mechanisms strictly connected to tumor growth. Moreover, mKI67, a marker of proliferation, is more expressed in cocultures with oC-MSCs than with yC-MSCs and this correlates with the increased proliferation rate observed in cocultures with MSCs derived from cervixes of old patients.

Finally, the secretion of IL2, IL6, IL10, IL12, IFN-*γ*, and TGF-*β* (that were differently produced by MSCs from old and young cervixes) was tested after cocultures with HeLa.

While cocultures did not affect the secretion of IL2, IL12, and IFN-*γ* compared to a mock sample, the level of IL6, IL10, and TGF-*β* were significantly increased after cocultures with “young” MSCs.

Only TGF-*β* displayed a significant increase in cocultures with oC-MSCs.

Cytokines mostly secreted after cocultures with yC-MSCs belong to the Th1–Th17 pathways, indicating as the presence of neoplastic cells increase the mechanism of the host's immune surveillance.

A previous study demonstrated that the expression of TGF-*β* decreased as tumor cells progressed from CIN to cervical carcinoma, indicating that the disruption of this signalling pathway might contribute to the malignant progression of cervical dysplasia [48]. In addition, under inflammatory conditions, TGF-*β* in the presence of IL6 drives the differentiation of T helper 17 (Th17) cells, which can promote further inflammation and augment autoimmune conditions [49]. The exact role of IL6 in CIN progression is still unclear; besides its increased expression reported by someone during cervical tumor development [50], there is the important role exerted as the master switch of acute inflammation.

Finally, IL10 plays a dual, controversial role in cervical carcinogenesis [51]; even if IL10 mRNA and/or protein are enhanced in several types of human cancer (including cervix), other works report that higher levels of IL10 may prevent cervical neoplasia by assisting in the elimination of HPV [52, 53]. IL10 enhances the proliferation and expression of immunologically important surface molecules and increases Th1 cytokine production. Actually, IL10 is used in combination with IL2 in the treatment of cervical cancer [54].

In conclusion, the present data suggest the following: (i) a mesenchymal stem cell (MSC) population persists inside the cervix; (ii) this cell population displays age-related properties; (iii) age may affect the regression rate of CIN by influencing the paracrine effect exerted by MSCs; and (iv) in the crosstalk with HeLa cells, MSCs derived from the cervixes of young patients play an antitumoral role. These results reinforce the evidence of the possibility of a conservative management of high-grade lesions (especially CIN2) in young women with a desire for future pregnancy, avoiding unnecessary treatments with the preservation of reproductive potential.

## Figures and Tables

**Table 1:** Primer sequences.

Gene symbol	Forward	Reverse
GAPDH	AGCCACATCGCTCAGACAC	GCCCAATACGACCAAATCC
RPLP0	CCATTCTATCATCAACGGGTACAA	TCAGCAAGTGGGAAGGTGTAATC
NANOG	TGAACCTCAGCTACAAACAG	CTGGATGTTCTGGGTCTGGT
SOX2	ACACCAATCCCATCCACACT	GCAAACTTCCTGCAAAGCTC
OCT4	AGCGAACCAGTATCGAGAAC	TTACAGAACCACACTCGGAC
KLF4	CCCACACAGGTGAGAAACCT	ATGTGTAAGGCGAGGTGGTC
cMET	TACCACTCCTTCCCTGCAAC	TCATTGCCCATTGAGATCAT
cFOS	AGAATCCGAAGGGAAAGGAA	CTTCTCCTTCAGCAGGTTGG
cJUN	TAACAGTGGGTGCCAACTCA	CCAAGTCCTTCCCACTCGT
mKi67	ACAGAAAAATCGAACTGGGAAA	GTTTATGAAGCCGATTCAGACC
MMP11	TCTCGTGGGTCCTGACTTCT	GTTGTCATGGTGGTTGTACCC
VEGF	CCTCCGAAACCATGAACTTT	ATGATTCTGCCCTCCTCCTTCT
CXCL12	TGAGAGCTCGCTTTGAGTGA	CACCAGGACCTTCTGTGGAT

**Table 2:** Immunophenotype of MSCs derived from the cervixes of young (yC-MSCs) and old (oC-MSCs) patients.

	yC-MSCs	oC-MSCs
HLA-DR	−	−
CD14	−	−
CD19	−	−
CD34	−	−
CD45	−	−
CD73	+	+
CD90	+	+
CD105	+	+
CD9	−	−

Positive immunolabelling (+) was defined as a level of fluorescence > 90% of the corresponding isotype-matched control antibodies. Percentages < 2% were considered negative (−). No differences were noted between the two subgroups.

## Data Availability

The data (PCR, ELISA, differentiation assay, cytofluorimetric assay, and proliferation) used to support the findings of this study are included within the article.
